# Efficacy and safety of avapritinib in advanced systemic mastocytosis: interim analysis of the phase 2 PATHFINDER trial

**DOI:** 10.1038/s41591-021-01539-8

**Published:** 2021-12-06

**Authors:** Jason Gotlib, Andreas Reiter, Deepti H. Radia, Michael W. Deininger, Tracy I. George, Jens Panse, Alessandro M. Vannucchi, Uwe Platzbecker, Iván Alvarez-Twose, Andrzej Mital, Olivier Hermine, Ingunn Dybedal, Elizabeth O. Hexner, Lisa K. Hicks, Lambert Span, Ruben Mesa, Prithviraj Bose, Kristen M. Pettit, Mark L. Heaney, Stephen T. Oh, Jayita Sen, Hui-Min Lin, Brenton G. Mar, Daniel J. DeAngelo

**Affiliations:** 1 Division of Hematology, Stanford Cancer Institute/Stanford University School of Medicine, Stanford, CA USA; 2 Department of Hematology and Oncology, University Hospital Mannheim, Heidelberg University, Mannheim, Germany; 3 Guy’s & St Thomas’ NHS Foundation Trust, London, UK; 4 Versiti Blood Research Institute and Division Hematology and Oncology, Medical College of Wisconsin, Milwaukee, WI USA; 5 ARUP Laboratories, University of Utah, Salt Lake City, UT USA; 6 Department of Oncology, Hematology, Hemostaseology and Stem Cell Transplantation, University Hospital RWTH Aachen, Aachen, Germany; 7 Center for Research and Innovation of Myeloproliferative Neoplasms – CRIMM, Azienda Ospedaliera Universitaria Careggi, University of Florence, Florence, Italy; 8 Leipzig University, Leipzig, Germany; 9 Institute of Mastocytosis Studies of Castilla-La Mancha, Spanish Reference Center of Mastocytosis, Toledo, Spain; 10 Department of Hematology and Transplantology, Medical University of Gdansk, Gdansk, Poland; 11 Department of Hematology, CEREMAST, Necker–Enfants Malades Hospital, APHP, and Imagine Institute, INSERM U1163, Paris University, Paris, France; 12 Department of Hematology, Oslo University Hospital, Oslo, Norway; 13 Abramson Cancer Center, University of Pennsylvania, Philadelphia, PA USA; 14 Division of Hematology/Oncology, St. Michael’s Hospital, University of Toronto, Toronto, Ontario Canada; 15 Department of Hematology, University Medical Center Groningen, University of Groningen, Groningen, the Netherlands; 16 Mays Cancer Center at UT Health San Antonio MD Anderson, San Antonio, TX USA; 17 The University of Texas MD Anderson Cancer Center, Houston, TX USA; 18 University of Michigan, Ann Arbor, MI USA; 19 Columbia University Medical Center, New York, NY USA; 20 Siteman Cancer Center at Barnes-Jewish Hospital and Washington University School of Medicine, St. Louis, MO USA; 21 Blueprint Medicines Corporation, Cambridge, MA USA; 22 Department of Medical Oncology, Dana-Farber Cancer Institute, Boston, MA USA

**Keywords:** Myeloproliferative disease, Myeloproliferative disease, Phase II trials, Mast cells

## Abstract

Advanced systemic mastocytosis (AdvSM) is a rare, *KIT* D816V-driven hematologic neoplasm characterized by mast cell infiltration and shortened survival. We report the results of a prespecified interim analysis of an ongoing pivotal single-arm phase 2 trial (no. NCT03580655) of avapritinib, a potent, selective KIT D816V inhibitor administered primarily at a once-daily starting dose of 200 mg in patients with AdvSM (*n* = 62). The primary endpoint was overall response rate (ORR). Secondary endpoints included mean baseline change in AdvSM–Symptom Assessment Form Total Symptom Score and quality of life, time to response, duration of response, progression-free survival, overall survival, changes in measures of disease burden and safety. The primary endpoint was successfully met (*P* = 1.6 × 10^-9^), with an ORR of 75% (95% confidence interval 57–89) in 32 response-evaluable patients with AdvSM who had sufficient follow-up for response assessment, including 19% with complete remission with full or partial hematologic recovery. Reductions of ≥50% from baseline in serum tryptase (93%), bone marrow mast cells (88%) and *KIT* D816V variant allele fraction (60%) were observed. The most frequent grade ≥3 adverse events were neutropenia (24%), thrombocytopenia (16%) and anemia (16%). Avapritinib demonstrated a high rate of clinical, morphological and molecular responses and was generally well tolerated in patients with AdvSM.

## Abstract (web summary)

In a prespecified interim analysis of a pivotal phase 2 trial, avapritinib, a selective KIT inhibitor, elicited robust clinical and molecular responses, was generally well tolerated and led to improved patient-reported outcomes in patients with advanced systemic mastocytosis.

## Main

Systemic mastocytosis (SM) is a rare hematologic neoplasm that is associated with the *KIT* D816V mutation in ~95% of cases. The *KIT* D816V mutation drives the increased proliferation and accumulation of neoplastic mast cells, leading to severe, debilitating and often unpredictable symptoms and poor quality of life (QoL)^1–3^. In AdvSM, mast cell infiltration leads to organ damage, referred to as ‘C-findings’ (that is, cytopenias or liver dysfunction), with limited treatment options and poor survival^2,4–6^. AdvSM is comprised of three subtypes: aggressive SM (ASM), SM with an associated hematologic neoplasm (SM-AHN) and mast cell leukemia (MCL)^2^. In SM-AHN, which represents 60–70% of cases of AdvSM^7,8^, patients concurrently have both SM and another World Health Organization-defined hematologic neoplasm, usually myeloid (for example, myelodysplastic syndrome, myeloproliferative neoplasm or an overlap)^9^. The *KIT* D816V mutation is frequently also present in cells comprising the AHN component^9^. Molecular subtyping of patients with AdvSM often reveals a heterogenous genetic landscape, with high-risk mutations in splicing factors, epigenetic regulators and transcription factors such as *SRSF2*, *ASXL1* and *RUNX1*, respectively^10–12^. Treatment options are very limited, with only the multikinase inhibitor midostaurin and, more recently, the selective KIT kinase inhibitor avapritinib approved for the treatment of AdvSM.

Avapritinib is a selective KIT and platelet-derived growth factor receptor-alpha (PDGFRA) kinase inhibitor with high potency for the KIT D816V and homologous PDGFRA-mutant proteins^1^. As detailed in the accompanying report^13^, avapritinib was investigated in patients with AdvSM in the phase 1 EXPLORER trial (no. NCT02561988)^14^. In this trial, avapritinib exhibited an ORR of 75% by modified (m)IWG–MRT–ECNM (International Working Group–Myeloproliferative Neoplasms Research and Treatment and European Competence Network) criteria, including 36% with complete remission with full (CR) or partial hematologic recovery (CRh), with a median follow-up of 23 months. Responses were seen at all starting doses (30–400 mg once daily (QD)) and deepened over subsequent cycles, but occurred most rapidly at 200 mg QD and higher. Patients experienced profound reductions in objective measures of mast cell burden, including complete molecular remission of *KIT* D816V, reversion of mast-cell-related organ damage and improvements in symptoms. Review of safety, rapid reduction of disease burden and response rate led to selection of 200 mg as the optimal dose for patients with AdvSM.

Here, we present the results of a prespecified interim analysis from the PATHFINDER trial^15^, an ongoing, international, multicenter, open-label, single-arm, phase 2 registrational trial (no. NCT03580655) of avapritinib 200 mg QD in adult patients with a centrally confirmed diagnosis of AdvSM. Patients were enrolled into cohort 1 (efficacy evaluable) if they had an evaluable mIWG–MRT–ECNM C-finding (that is, cytopenias, liver function abnormalities, splenomegaly, ascites, pleural effusion) (Supplementary Table 1) at baseline, or MCL irrespective of the presence of C-findings. The primary endpoint of the trial was ORR, including CR/CRh, partial remission (PR) and clinical improvement (CI), as per mIWG–MRT–ECNM criteria (Supplementary Table 2), which was tested against 28%, the ORR of midostaurin, as per IWG–MRT–ECNM criteria^16^. A prespecified interim analysis was performed when 32 patients in cohort 1 had sufficient follow-up for response evaluation (interim analysis efficacy population). In addition, cohort 2 included patients without an evaluable C-finding at baseline (that is, evaluable C-findings that resolved with previous therapy or nonevaluable C-findings such as weight loss or large osteolytic lesions) and were therefore not evaluable for response. All enrolled patients (safety population) were included in secondary endpoints, which included changes in patient symptoms, reduction in measures of disease burden and safety.

## Results

### Participants

**Extended Data Fig. 1:**
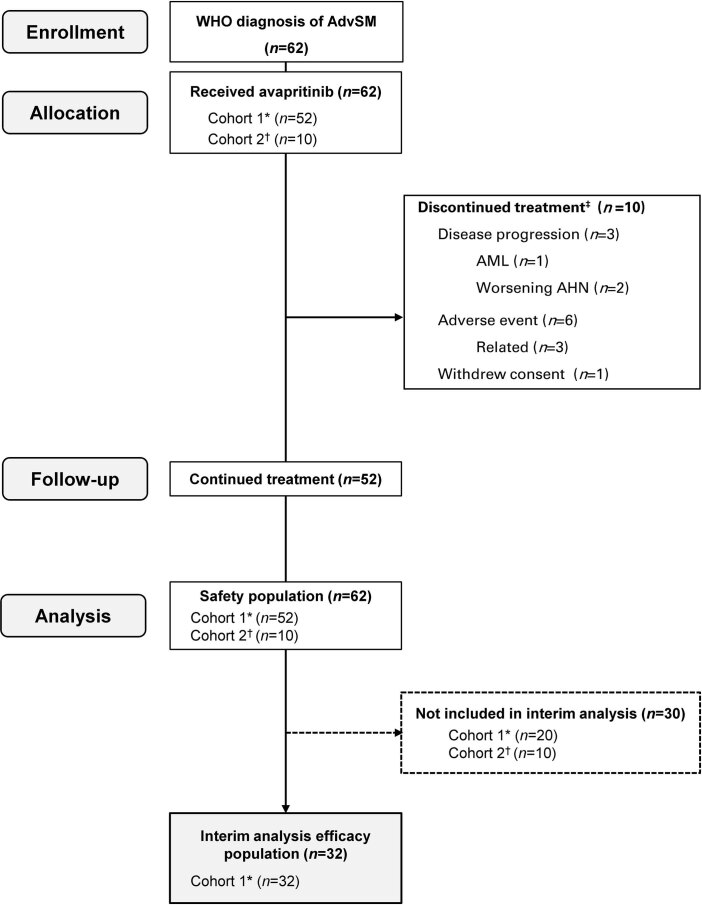
Patient Disposition. *Cohort 1: Patients with AdvSM and ≥1 mIWG-MRT-ECNM criteria for evaluable disease (have severe and quantifiable organ damage (an evaluable C-finding) or have MCL (regardless of C-findings)) as confirmed by the SSC. Twenty patients in Cohort 1 were not included in the interim analysis efficacy population due to lack of sufficient follow-up. ^†^Patients with AdvSM who were not considered eligible for an adjudicated response and were confirmed centrally to have ASM or SM-AHN, but were lacking an evaluable C-finding as determined by the SSC. ^‡^No patient discontinued from treatment due to lost to follow-up, protocol deviation, pregnancy, investigator decision, non-compliance, sponsor decision, or other reason. AdvSM, advanced systemic mastocytosis; AHN, associated hematologic neoplasm; AML, acute myeloid leukemia; ASM, aggressive systemic mastocytosis; MCL, mast cell leukemia; mIWG-MRT-ECNM, modified International Working Group-Myeloproliferative Neoplasms Research and Treatment and European Competence Network on Mastocytosis; SM-AHN, systemic mastocytosis with an associated hematologic neoplasm; SSC, study steering committee; WHO, World Health Organization.

Between 21 November 2018 and 23 June 2020, 62 patients with prospectively centrally adjudicated AdvSM were enrolled (ASM (*n* = 9), SM-AHN (*n* = 43) and MCL (*n* = 10)) and received avapritinib primarily at a starting dose of 200 mg QD (*n* = 60; two patients started at 100 mg QD), across cohort 1 (*n* = 52) and cohort 2 (*n* = 10) (Extended Data Fig. 1). Avapritinib was administered continuously in 28-day cycles until progression, intolerance, withdrawal by the investigator or patient or death.

**Extended Data Fig. 2:**
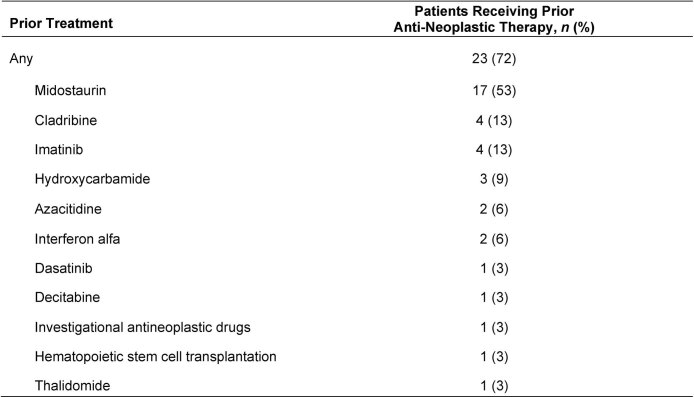
Prior Anti-Neoplastic Therapies (Interim Analysis Efficacy Population, *n* = 32). Table shows details of prior therapies received by patients that ended on or before the first dose date of avapritinib.

**Extended Data Fig. 3:**
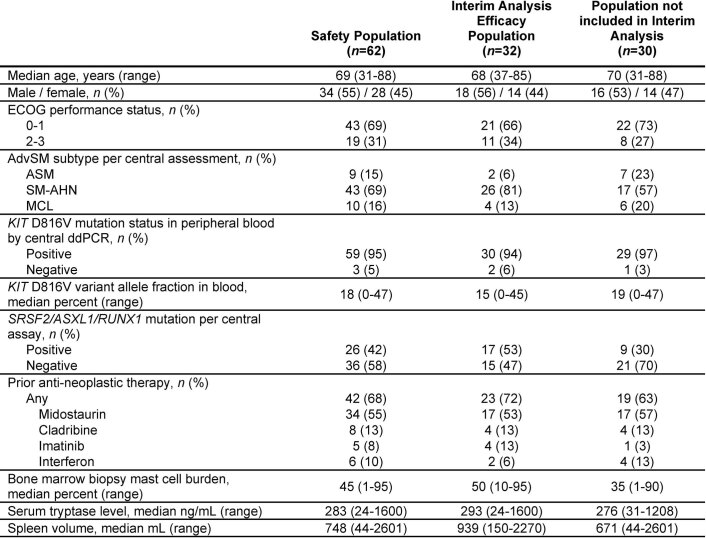
Baseline Characteristics. Baseline characteristics including for patients not included in the interim analysis (*n* = 30). AdvSM, advanced systemic mastocytosis; ASM, aggressive systemic mastocytosis; ddPCR, droplet digital polymerase chain reaction; ECOG, Eastern Cooperative Oncology Group; MCL, mast cell leukemia; mIWG-MRT-ECNM, modified International Working Group-Myeloproliferative Neoplasms Research and Treatment and European Competence Network on Mastocytosis; SM-AHN, systemic mastocytosis with associated hematologic neoplasm.

**Extended Data Fig. 4:**
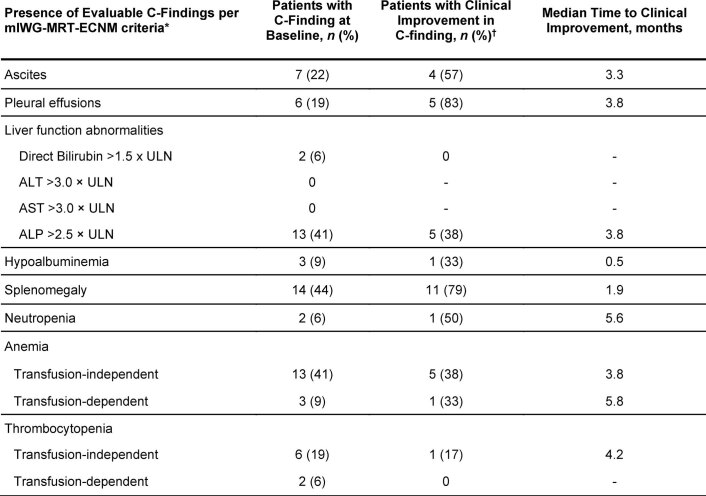
Response by mIWG-MRT-ECNM C-Findings (Interim Analysis Efficacy Population, n = 32). *Definitions of C-findings per mIWG-MRT-ECNM and criteria for measuring clinical improvement are presented in Supplementary Table 1 and Supplementary Table 2. ^†^Percentages are based on the number of patients with C-finding at baseline. ALP, alkaline phosphatase; ALT, alanine aminotransferase; AST, aspartate aminotransferase; mIWG-MRT-ECNM, modified International Working Group-Myeloproliferative Neoplasms Research and Treatment and European Competence Network on Mastocytosis; ORR, overall response rate; ULN, upper limit of normal.

The interim analysis was triggered when 32 response-evaluable patients in cohort 1 achieved sufficient follow-up for confirmed evaluation of response. The median age was 68 years (range, 37–85) and 56% of patients were male (Table 1). By central assessment, 94% were positive for the *KIT* D816V mutation and 53% carried an additional mutation in at least one of the genes *SRSF2*, *ASXL1* or *RUNX1* (*S/A/R*), which is associated with poor survival in SM^12^. The majority of patients (72%) had received previous antineoplastic therapy, including 53% with midostaurin (Extended Data Fig. 2). Baseline median bone marrow mast cell percentage was 50% (range, 10–95) and median serum tryptase was 293 ng ml^–1^ (range, 24–1,600). The median *KIT* D816V variant allele fraction (VAF) in peripheral blood (which rarely has circulating mast cells) was 15% (range, 0–45). Median spleen volume was 939 ml (range, 150–2,270). Baseline characteristics were generally balanced between the safety population and those included versus not included in the interim analysis efficacy population (Extended Data Fig. 3). In the interim analysis efficacy population, the most common baseline eligible mIWG–MRT–ECNM C-findings (Supplementary Table 1) were splenomegaly (44%), elevated alkaline phosphatase (41%) and transfusion-independent anemia (41%) (Extended Data Fig. 4).

**Table 1:** Baseline characteristics

	Safety population (*n* = 62)	Interim analysis efficacy population (*n* = 32)
Median age, years (range)	69 (31–88)	68 (37–85)
Male/female, *n* (%)	34 (55)/28 (45)	18 (56)/14 (44)
ECOG performance status, *n* (%)		
0–1	43 (69)	21 (66)
2–3	19 (31)	11 (34)
AdvSM subtype as per central assessment, *n* (%)		
ASM	9 (15)	2 (6)
SM-AHN	43 (69)	26 (81)
MCL	10 (16)	4 (13)
*KIT* D816V mutation status in peripheral blood by central ddPCR, *n* (%)		
Positive	59 (95)	30 (94)
Negative	3 (5)	2 (6)
*KIT* D816V variant allele fraction in blood, median percentage (range)	18 (0–47)	15 (0–45)
*SRSF2/ASXL1/RUNX1* mutation as per central assay, *n* (%)		
Positive	26 (42)	17 (53)
Negative	36 (58)	15 (47)
Previous antineoplastic therapy, *n* (%)		
Any	42 (68)	23 (72)
Midostaurin	34 (55)	17 (53)
Cladribine	8 (13)	4 (13)
Imatinib	5 (8)	4 (13)
Interferon	6 (10)	2 (6)
Bone marrow biopsy mast cell burden median percentage (range)	45 (1–95)	50 (10–95)
Serum tryptase level, median ng ml^–1^ (range)	283 (24–1,600)	293 (24–1,600)
Spleen volume, median ml (range)	748 (44–2,601)	939 (150–2,270)

ddPCR, droplet digital PCR.

### Interim analysis of efficacy

**Fig. 1:**
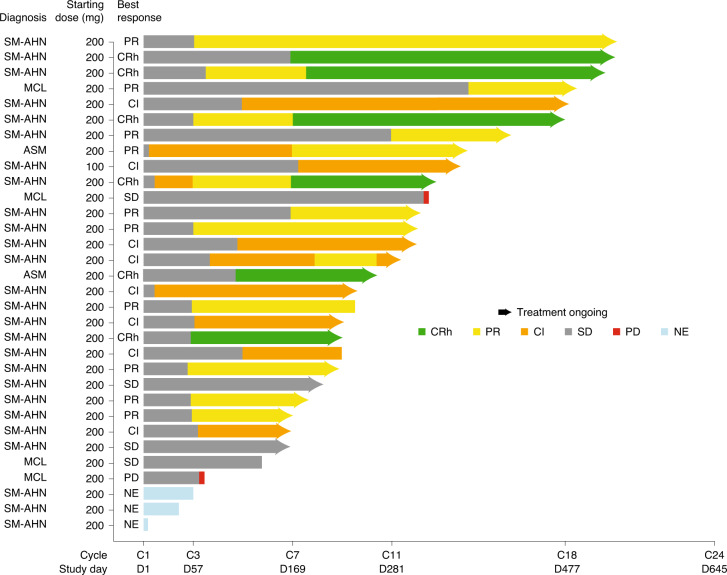
Adjudicated mIWG–MRT–ECNM response by cycle. Includes patients in the interim analysis efficacy population (*n* = 32). SD, stable disease; PD, progressive disease; NE, not evaluable.

Among 32 patients in the interim analysis efficacy population (median follow-up of 10.4 months), the confirmed ORR (CR/CRh/PR/CI; primary endpoint) was 75% (*n* = 24, 95% confidence interval, 57–89, *P* = 1.6 × 10^-9^), with six patients (19%) achieving CRh. Ten patients (31%) achieved PR and eight (25%) had CI (Table 2). Responses were observed in all AdvSM subtypes, regardless of exposure to previous therapy (Table 2). Additionally, ORR was similar among patients with (71% (95% confidence interval, 44–90), 12/17) and without (80% (95% confidence interval, 52–96), 12/15) baseline *S*/*A*/*R* mutations. Responses were rapid, with a median time to response of 2 months (range, 0.3–12.2). Responses continued to improve over time (Fig. 1), with a median time to CRh of 5.6 months (range, 1.8–6.1).

**Table 2:** Response rates by adjudicated mIWG–MRT–ECNM response criteria

Best confirmed response, *n* (%)	All AdvSM (*n* = 32)	AdvSM subtype			Previous therapy		Previous midostaurin	
		ASM (*n* = 2)	SM-AHN (*n* = 26)	MCL (*n* = 4)	Yes (*n* = 23)	No (*n* = 9)	Yes (*n* = 17)	No (*n* = 15)
ORR (CR + CRh + PR + Cl)	24 (75)	2 (100)	21 (81)	1 (25)	17 (74)	7 (78)	14 (82)	10 (67)
95% Confidence interval	57–89	16–100	61–93	1–81	52–90	40–97	57–96	38–88
Best response								
CR or CRh	6 (19)	1 (50)	5 (19)	0	3 (13)	3 (33)	3 (18)	3 (20)
CR	0	0	0	0	0	0	0	0
CRh	6 (19)	1 (50)	5 (19)	0	3 (13)	3 (33)	3 (18)	3 (20)
PR	10 (31)	1 (50)	8 (31)	1 (25)	7 (30)	3 (33)	5 (29)	5 (33)
CI	8 (25)	0	8 (31)	0	7 (30)	1 (11)	6 (35)	2 (13)
Stable disease	4 (13)	0	2 (8)	2 (50)	2 (9)	2 (22)	0	4 (27)
Progressive disease	1 (3)	0	0	1 (25)	1 (4)	0	0	1 (7)
Not evaluable	3 (9)^a^	0	3 (12)	0	3 (13)	0	3 (18)	0

^a^Three (9%) patients were in the interim analysis efficacy population but were assessed as not evaluable for response due to coming off trial before a confirmed response could be determined (13 weeks).

**Fig. 2:**
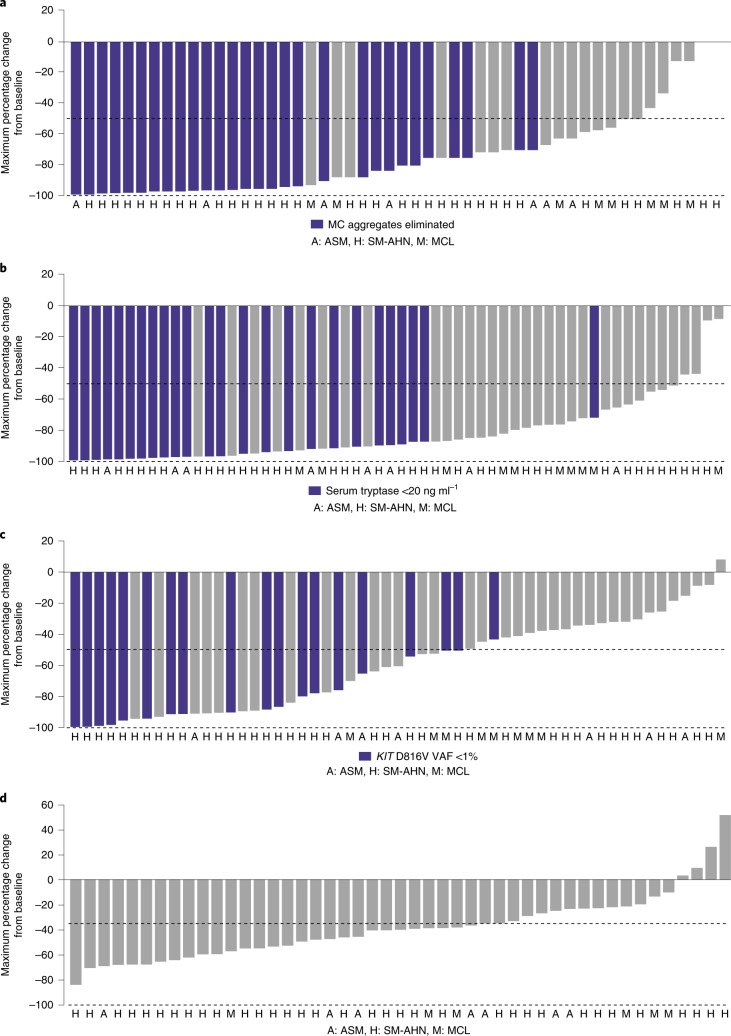
Change from baseline in clinicopathological measures of response. **a**, Bone marrow mast cells. **b**, Serum tryptase. **c**, *KIT* D816V variant allele fraction. **d**, Spleen volume. MC, mast cell.

In the safety population (*n* = 62), consistent and profound reductions in measures of mast cell burden (secondary endpoint) were observed in all enrolled patients with baseline and postbaseline assessments. A reduction of ≥50% in bone marrow mast cells was observed in 88% (44/50) of patients, and 60% (30/50) had elimination of bone marrow mast cell aggregates (Fig. 2a). The serum tryptase level decreased by ≥50% in 93% (54/58) of patients, and 43% (25/58) of patients achieved serum tryptase levels <20 ng ml^–1^ (Fig. 2b).

**Extended Data Fig. 5:**
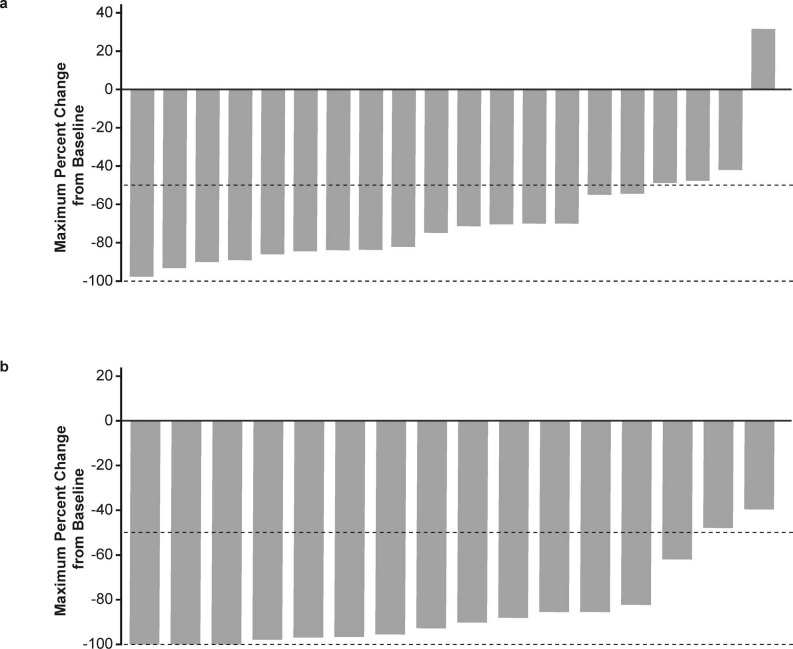
Change from Baseline in Clinicopathological Measures of Disease Burden. **a**, Peripheral blood absolute monocyte count in patients with SM-CMML. **b**, Peripheral blood absolute eosinophil count in patients with baseline eosinophilia. SM-CMML, systemic mastocytosis with chronic myelomonocytic leukemia.

Profound reductions in disease activity beyond mast cells were also observed, with a decrease of ≥50% in absolute monocyte counts in 80% (16/20) of patients with SM and chronic myelomonocytic leukemia (SM-CMML), and a decrease of ≥50% in absolute eosinophil counts in 88% (14/16) of patients with eosinophilia, including all three patients with SM and chronic eosinophilic leukemia (SM-CEL) (Extended Data Fig. 5). Consistent with efficacy against *KIT* D816V mutant-bearing cells, there was a substantial (≥50%) reduction in *KIT* D816V VAF in the peripheral blood in 60% (33/55) of patients; 35% (19/55) of patients achieved a VAF of <1% (Fig. 2c). Spleen volume, which may be greatly enlarged due to involvement by both neoplastic mast cells and AHN, was reduced from baseline by ≥35% in 66% (31/47) of patients (Fig. 2d).

The majority of mIWG–MRT–ECNM C-findings in patients in the interim analysis efficacy population resolved from baseline, including 83% of pleural effusions, 79% of splenomegaly and 57% of ascites (Extended Data Fig. 4). Resolution of cytopenia was less frequent, despite elimination of bone marrow mast cell aggregates in the majority of patients, consistent with additional etiologies for cytopenia, such as persistent AHN and/or avapritinib-related myelosuppression.

Median progression-free survival (PFS) in the interim analysis efficacy population and median overall survival (OS) in the safety population (secondary endpoints) had not been reached at the time of data cutoff. The estimated 6-, 9- and 12-month PFS rates were 91, 87 and 79%, respectively; corresponding OS rates were 94, 86 and 86%.

### Safety

As of the data cutoff, 52 (84%) of 62 patients were still on treatment with a median follow-up of 7.0 months (range 5.6–8.1). Reasons for treatment discontinuation included adverse events (AEs) in six patients (with three considered treatment-related according to the local site investigator (decreased weight, subdural hematoma and bleeding propensity with subcutaneous hematoma)), disease progression as per the investigator in three patients (with one transformation to acute myeloid leukemia and two with worsening AHN) and consent withdrawal in one patient (Extended Data Fig. 1).

The most frequent AEs are presented in Table 3. The most frequent nonhematologic AEs (any grade; grade ≥3) were peripheral edema (50%; 3%), periorbital edema (48%; 3%), diarrhea (23%; 2%), nausea (18%; 2%) and vomiting (18%; 2%). The most frequent hematologic AEs (any grade; grade ≥3) were thrombocytopenia (45%; 16%), anemia (32%; 16%) and neutropenia (24%; 24%), although grade 4 neutropenia (absolute neutrophil count <0.5 × 10^9^ l^–1^) was uncommon, at 8%. Grade ≥3 treatment-related AEs were reported in 32 (52%) patients, the most frequent of which were neutropenia (23%) and thrombocytopenia (15%). There were three (5%) deaths due to AEs (disease progression, necrotizing fasciitis and hemorrhagic shock), none of which were considered related to treatment. Cognitive effects (confusional state, memory impairment and cognitive disorder) occurred in seven (11%) patients (Table 3) and were primarily grade 1 (*n* = 6), with one grade 2.

**Table 3:** Adverse events (safety population, *n* = 62)

	Any-cause AEs		Treatment-related AEs	
	Any grade	Grade ≥3	Any grade	Grade ≥3
Any AE, *n* (%)	62 (100)	42 (68)	57 (92)	32 (52)
Nonhematologic AEs^a^, *n* (%)				
Peripheral edema	31 (50)	2 (3)	26 (42)	1 (2)
Periorbital edema^b^	30 (48)	2 (3)	28 (45)	2 (3)
Diarrhea	14 (23)	1 (2)	7 (11)	1 (2)
Nausea	11 (18)	1 (2)	5 (8)	0
Vomiting	11 (18)	1 (2)	6 (10)	1 (2)
Fatigue	9 (15)	2 (3)	6 (10)	2 (3)
Increased blood alkaline phosphatase	7 (11)	3 (5)	2 (3)	1 (2)
Hematologic AEs^a^, *n* (%)				
Thrombocytopenia^b^	28 (45)	10 (16)	25 (40)	9 (15)
Anemia^b^	20 (32)	10 (16)	12 (19)	5 (8)
Neutropenia^b^	15 (24)	15 (24)^c^	14 (23)	14 (23)^c^
Leukopenia^b^	7 (11)	3 (5)	7 (11)	3 (5)
AEs of special interest, *n* (%)				
Cognitive effects	7 (11)	0	–	–
Confusional state	3 (5)	0	–	–
Memory impairment	3 (5)	0	–	–
Cognitive disorder	2 (3)	0	–	–
Intracranial bleeding	1 (2)	1 (2)^d^	–	–
Subdural hematoma	1 (2)	1 (2)^d^	–	–

^a^Any-cause AEs in ≥15% (any grade) or ≥5% (grade ≥3) of patients are listed.

^b^Pooled terms.

^c^Grade 4 neutropenia of any cause and treatment-related occurred in 5 (8%) and 4 (6%) patients, respectively.

^d^Grade 4 event that occurred before risk mitigation measures were implemented.

There was one (1.6%) intracranial bleeding (ICB) event (subdural hemorrhage), in a patient with severe thrombocytopenia at baseline (platelets, 49 × 10^9^ l^–1^) who was enrolled before the exclusion (below) of such patients. The patient was treated with avapritinib despite worsening thrombocytopenia (platelets, 33 × 10^9^ l^–1^) and developed a grade 2 subdural hematoma 2 days after a single dose of enoxaparin. The patient was not taking antiplatelet agents and had a relatively normal international normalized ratio and activated partial thromboplastin time of 1.2 and 28.1 s, respectively, before the ICB event. The patient subsequently recovered from the event and was retreated with avapritinib due to continuing benefit. However, the patient subsequently developed a grade 4 subdural hematoma despite platelets being 167 × 10^9^ l^–1^, leading to treatment discontinuation.

Safety analysis of this event and similar ICB events in the concurrent phase 1 trial identified patients with baseline severe thrombocytopenia at substantially increased risk of ICB; therefore, patients with platelets <50 × 10^9^ l^–1^ at baseline were subsequently excluded from enrollment in both studies. In addition, the studies were amended to include increased platelet count monitoring, updated dose guidance for interruption, support for severely low platelet counts and treatment discontinuation for any grade of ICB events.

**Extended Data Fig. 6:**
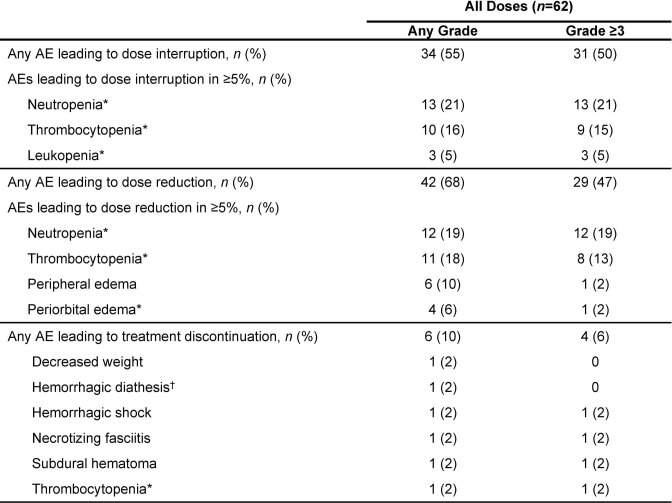
Adverse Events Leading to Dose Modifications. *Pooled terms. ^†^Patient reported to have increased bleeding propensity with subcutaneous hematoma. AE, adverse event.

In total, six patients (10%) experienced AEs leading to permanent treatment discontinuation. Two (5%) patients discontinued due to a treatment-related serious AE (subdural hematoma and bleeding propensity with subcutaneous hematoma). Dose interruptions due to AEs occurred in 34 (55%) patients, most commonly due to neutropenia (21%) and thrombocytopenia (16%) (Extended Data Fig. 6). AEs led to dose reductions in 42 (68%) patients, most commonly due to neutropenia (19%) and thrombocytopenia (18%). The median time to dose reduction was 7.4 weeks, and most patients were taking a daily dose of 100 mg after cycle 3. The median daily dose was 138 mg (range, 38–240), consistent between patients with or without before exposure to therapy or midostaurin.

### Patient-reported outcomes and QoL

Patients’ baseline QoL was negatively impacted by their disease, with a pretreatment mean European Organization for Research and Treatment of Cancer Core QoL Questionnaire C30 (EORTC–QLQ–C30–QoL) score of only 37.8 (range, 0–100, where 0 represents the lowest QoL and 100 the highest) and a high proportion of patients (31%) with a poor Eastern Cooperative Oncology Group (ECOG) performance status of 2 or 3. Mean and median Patient Global impression of Symptom Severity (PGIS) scores were 2.6 and 3.0, respectively (where 0 represents no symptoms and 4 very severe symptoms). The AdvSM–Symptom Assessment Form (SAF), an AdvSM-specific patient reported outcomes tool, showed that fatigue, abdominal pain and spots were the most severe symptoms, with a mean total symptom score (TSS) of 18.3, which was the sum of eight possible common symptoms (each scored 0–10, where 0 represents no symptoms and 10 is the worst imaginable). At baseline, patients had frequent supportive care medication use, including H1 antihistamines (58%), H2 antihistamines (39%) and corticosteroids (32%). Patients on corticosteroids remained evaluable if their dose did not exceed 20 mg d^–1^ of prednisone or equivalent.

**Fig. 3:**
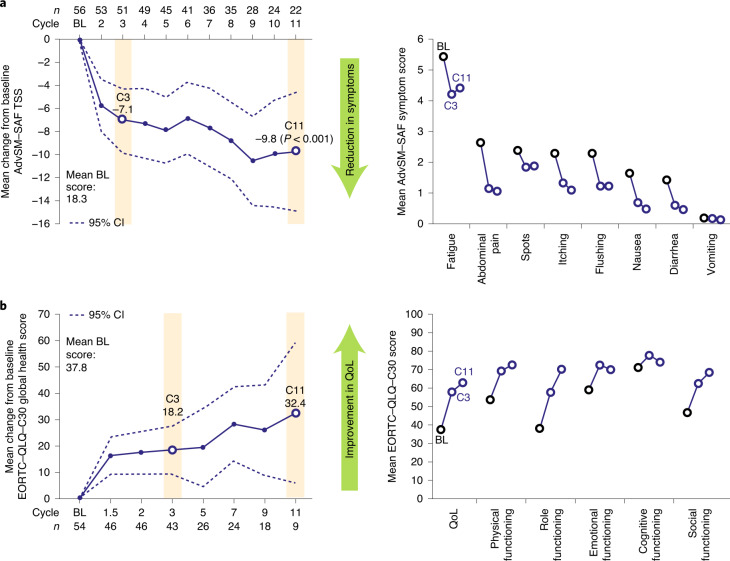
Patient-reported outcomes. **a**, AdvSM–SAF TSS. **b**, EORTC–QLQ–C30 global health score. BL, baseline. C, cycle.

Patient-reported symptoms, as measured by TSS score, improved rapidly following treatment initiation, dropping by 7.1 points from baseline at cycle 3 (*n* = 51) and by 9.8 points from baseline at treatment cycle 11 (*n* = 22; *P* < 0.001) (secondary endpoint; Fig. 3a). Mean symptom scores were lower than baseline at cycles 3 and 11 for all SM symptoms, including fatigue, abdominal pain, spots, itching, flushing, nausea, diarrhea and vomiting. Mean and median PGIS scores improved to 1.6 and 2.0 (moderate symptoms that are difficult to ignore), respectively, by cycle 3 and to 1.2 and 1.0 (minimal symptoms that are easy to ignore), respectively, by cycle 11 (secondary endpoint).

Quality of life, as assessed by EORTC–QLQ–C30, improved on trial with noteworthy improvements in physical (strenuous activity), role (work or household jobs), emotional (irritability, feeling tense and depression), cognitive (memory and concentration) and social (family life and social activities) functioning domains (secondary endpoint; Fig. 3b).

## Discussion

This prespecified interim analysis of the phase 2 PATHFINDER trial demonstrated that avapritinib at a starting dose of 200 mg QD exhibited clinical benefit in patients with AdvSM, confirming findings from the phase 1 EXPLORER trial. In total, 75% of patients achieved a response regardless of AdvSM subtype, previous therapy or adverse *S*/*A*/*R* comutations, with a rapid median time to response of 2 months. With median follow-up of 10.4 months, 19% of patients had normalized all baseline evaluable C-findings, eliminated mast cell aggregates and reduced tryptase to <20 ng ml^–1^, to achieve CRh. In June 2021, data from this and the phase 1 EXPLORER trial (no. NCT02561988) formed the basis of approval by the United States Food and Drug Administration for the treatment of adults with AdvSM at a recommended starting dose of 200 mg orally QD.

Consistent and profound reductions in mast cell burden (bone marrow mast cells, serum tryptase) and normalization of SM-related organ damage (that is, liver function abnormalities, ascites, spleen size) were observed in patients across all AdvSM subtypes regardless of previous therapy. Although improvements in cytopenias were seen in some patients, including improvement of transfusion-dependent anemia in one patient, cytopenias were less likely to normalize than other C-findings, even in patients who no longer had bone marrow mast cell aggregates. This implies that persistent cytopenias were related to non-mast cell causes, such as drug effect and/or the remaining AHN component.

In addition, avapritinib demonstrated evidence of broader disease activity in patients with SM-AHN, which comprises the majority of AdvSM variants. For example, substantial reductions of monocytosis in 80% of patients with SM-CMML and eosinophilia in 88% patients with baseline eosinophilia or SM-CEL were observed. Overall, 60% of patients had substantial reductions in peripheral blood *KIT* D816V VAF, consistent with broad activity against both *KIT* D816V-positive mast cells (which rarely circulate in the blood) and cells derived from the AHN which often harbor *KIT* D816V, reflecting multilineage involvement of the mutation. Some patients with SM-AHN may need AHN therapy in combination or in sequence with a KIT-inhibitor-based regimen to address the genetic and biologic heterogeneity which underpins this AdvSM subtype. However, most patients had improvement in their AHN with avapritinib alone. This activity supports exploration of avapritinib in other *KIT* D816V-positive hematologic malignancies.

Patients had marked impairments in QoL at baseline due to their AdvSM symptoms yet reported rapid, durable and substantial reductions from baseline in every AdvSM symptom assessed, and improvements in QoL and functional impairment. Median global symptom severity as measured by PGIS had improved from severe to minimal by cycle 11.

Avapritinib was generally well tolerated, with few discontinuations due to AEs. Overall, the safety profile at the starting dose of 200 mg QD in the PATHFINDER trial included mostly low-grade fluid retention and gastrointestinal symptoms as the most common nonhematologic AEs. Cytopenias were the most common grade ≥3 AEs and reason for dose reduction, most commonly in patients with baseline cytopenias, and 100 mg was the median daily dose after cycle 3. Cognitive effects, which are dose-related, were relatively uncommon at a starting dose of 200 mg.

The incidence of ICB was low (1.6%), and similar to that observed with avapritinib in patients with GIST^17,18^, probably due to mitigation steps for severe thrombocytopenia which were implemented early in enrollment. These included exclusion of patients with platelets <50 × 10^9^ l^–1^, closer monitoring of platelet counts, stricter dose modification guidelines and use of platelet transfusions and growth factors to maintain platelet count to ≥50 × 10^9^ l^–1^. Dose-related, low-grade cognitive events were observed but did not result in substantial dose reductions. The optimal starting dose of 200 mg QD was confirmed in the current trial, because preliminary data generated in the EXPLORER trial suggested that this dose maintained efficacy while improving tolerability.

These data reinforce KIT D816V as a clinically validated drug target in AdvSM. While the multikinase inhibitor midostaurin is already approved for use in patients with AdvSM, there remains a substantial need for improvement in the overall rate, durability and quality of responses^19–22^. Treatment with midostaurin for AdvSM can lead to challenging gastrointestinal side effects, limiting its efficacy. With avapritinib, a potent, selective inhibitor of KIT D816V, the clinical aim in AdvSM is to achieve deeper and enduring clinical, morphological and molecular responses, which could translate into extended survival with improved QoL.

A potential limitation of this prespecified interim analysis is the short duration of follow-up (10.4 months) in a subset of trial patients, which will be addressed in long-term follow-up analyses of the study. It is anticipated that findings from long-term analyses will be consistent with those observed in the phase 1 trial^13^.

In conclusion, interim results from the current phase 2 PATHFINDER trial corroborate the promising and durable outcomes observed in the phase 1 EXPLORER trial. Avapritinib administered at a starting dose of 200 mg QD was well tolerated and led to profound reductions in disease burden, improved patient symptoms and QoL, and elicited deep molecular responses of *KIT* D816V, highlighting the potential for modification of AdvSM disease natural history.

## Methods

### Participants

Eligible patients were ≥18 years of age and with a diagnosis of AdvSM confirmed by central review. The primary efficacy endpoint of ORR as per mIWG–MRT–ECNM criteria^23^ required an evaluable baseline C-finding (Supplementary Table 1), and these patients were enrolled into cohort 1. Patients who did not have an evaluable C-finding at baseline could not be assessed for the primary endpoint and were enrolled into cohort 2, with the exception of patients with MCL who, as per criteria, were enrolled in cohort 1 and evaluated for reductions in disease burden. As a result of emerging evidence that an increased risk of ICB is associated with grade 3 thrombocytopenia, the protocol was amended to exclude patients with baseline platelet count <50 × 10^9^ l^–1^.

Additional inclusion criteria were as follows: patients with SM-AHN who had received previous treatment for the AHN component of disease if, in the opinion of the investigator, such therapy was appropriate; bone marrow biopsy taken within 56 days of cycle 1, day 1 (C1D1); serum tryptase levels ≥20 ng ml^–1^; ECOG performance status 0–3; discontinued cytoreductive therapy due to disease progression, refractory disease, lack of efficacy or intolerance if receiving therapy within the preceding 12 weeks; stable dose of nonantineoplastic SM therapies or corticosteroids (≤20 mg d^–1^ prednisone or equivalent) for ≥14 days before C1D8; and able to provide written informed consent. Patients were excluded for the following reasons: received previous treatment with avapritinib; received any cytoreductive therapy or an investigational agent <14 days and, for cladribine, interferon alpha, pegylated interferon and any antibody therapy, <28 days before obtaining screening bone marrow biopsy; received previous radiotherapy or any hematopoietic growth factor within 14 days before screening bone marrow biopsy; requires concomitant medication that is a strong inhibitor, strong inducer or moderate inducer of cytochrome P450 3A4; had a major surgical procedure within 14 days of the first dose of study drug; is a candidate for allogeneic hematopoietic stem cell transplantation for treatment of SM; eosinophilia and known positivity for the *FIP1L1–PGDFRA* fusion, unless the patient has demonstrated relapse or progressive disease on previous imatinib therapy; has history of another primary malignancy (within 3 years before the first dose of study drug), cerebrovascular accident or transient ischemic attacks (within 1 year before the first dose of study drug), or seizure disorder; has abnormal laboratory findings or QT interval corrected using Fridericia’s formula >480 ms; has known risk or recent history of ICB; has a primary brain malignancy or metastases to the brain; has clinically significant, uncontrolled cardiovascular disease; unless postmenopausal (females) or surgically sterile; is unwilling to abstain from sexual intercourse or employ highly effective contraception from the first dose of study drug and for at least 6 weeks after the last dose of study drug; pregnant or breastfeeding; hypersensitivity to avapritinib or to any of the excipients; unwilling or unable to comply with the study procedures or requirements; and participation in another interventional study.

### Trial design and treatment

PATHFINDER (ClinicalTrials.gov identifier no. NCT03580655) is an ongoing, international, multicenter, open-label, single-arm, phase 2 registrational trial conducted in North America and Europe. Avapritinib was administered at a starting dose of 200 mg QD in 28-day cycles until progression, intolerance, withdrawal by the investigator or patient or death. Dose modification to as low as 25 mg QD was allowed as per prespecified criteria. Dose increases to 300 mg QD were allowed for lack of efficacy and dose interruptions for platelet counts <50 × 10^9^ l^–1^ were required, although platelet transfusion and growth factor support were allowed on trial.

Previous cytoreductive therapy or investigational agents were permitted if received up to 14 days before the screening bone marrow sample or up to 28 days (cladribine, interferon alpha, pegylated interferon or any antibody therapy) before the screening marrow biopsy. Medications, including palliative and supportive care for disease-related symptoms, were permitted during the study and may include the following classes of agents: histamine receptor H1 and H2 blockers; proton pump inhibitors; osteoclast inhibitors (that is, bisphosphonates); leukotriene receptor antagonists; corticosteroids (not exceeding 20 mg d^–1^ of prednisone or equivalent, and dose must be stable for ≥14 days before C1D8); cromolyn sodium and other mast cell stabilizers; and omalizumab.

### Trial outcomes and assessments

The primary endpoint was optimal ORR as per mIWG–MRT–ECNM criteria (Supplementary Table 2). Modifications to the IWG–MRT–ECNM criteria were previously described^7,13^. Responses as per mIWG–MRT–ECNM criteria were adjudicated by the Study Steering Committee based on data from every trial visit, and required confirmation of ≥12 weeks. Bone marrow samples were assessed by central pathology, and serum tryptase and *KIT* D816V VAF in the blood were analyzed by central laboratory.

The primary analysis was originally planned after 63 patients in cohort 1 had received at least ten cycles of therapy but, due to the high efficacy observed in the EXPLORER trial, an interim analysis was introduced early in the PATHFINDER trial, to be triggered when 32 patients in cohort 1 (interim analysis efficacy population) had sufficient follow-up to be adjudicated for response as per mIWG–MRT–ECNM criteria.

The key secondary endpoint was mean change from baseline in patient-reported TSS of the AdvSM–SAF, a validated measure to assess treatment benefit in AdvSM patients^24,25^. Other secondary endpoints included time to response (time from the start of treatment to the response according to mIWG–MRT–ECNM criteria); duration of response (DOR; time from first documented response to the date of the first documented disease progression/loss of response or death due to any cause, whichever occurred first); PFS (time from the start of treatment to the date of the first documented disease progression as per mIWG–MRT–ECNM criteria or death due to any cause, whichever occurred first); OS (time from the start of treatment to the date of death); and changes in mast cell burden, safety and QoL using EORTC–QLQ–C30. Additional planned secondary endpoints not reported in this manuscript included pharmacokinetics of avapritinib and morphological response based on the Pure Pathological Response criteria^7,26^.

Response according to mIWG–MRT–ECNM criteria was assessed at C1D15, C2D1, C3D1, C7D1 and every six cycles thereafter, 12 weeks after documentation of CR or PR, 4 weeks after progressive disease of the AdvSM and/or AHN components and at the end of therapy if discontinued for a reason other than progressive disease or initiation of alternative cytoreductive therapy. Patient-reported outcomes (AdvSM–SAF, PGIS and EORTC–QLQ–C30) were collected at each visit through cycle 17 and at the end of therapy (if before or at cycle 17).

All patients were followed for safety (until 30 days after treatment discontinuation) and for long-term survival every 3 months. Safety assessments included determination of ECOG performance status, clinical laboratory testing, vital signs, electrocardiograms, brain imaging (magnetic resonance imaging or computerized tomography scan) and physical examinations. Treatment-emergent AEs were defined as any AE that occurred between the first dose of avapritinib through 30 days after the last dose of avapritinib, and were graded according to National Cancer Institute Common Terminology Criteria for AEs, v.5.0.

### Statistical analysis

The null hypothesis ORR of 28% versus the alternative hypothesis, ORR of 50%, a one-sided type I error rate of 0.025 and a sample size of approximately 63 patients in cohort 1 were determined to have 93.5% power using the exact one-sample binomial test. The null hypothesis, ORR of 28%, was based on ORR as per IWG–MRT–ECNM criteria for midostaurin^19^. The data cutoff date for this interim analysis was 23 June 2020, and was performed when 32 patients in cohort 1 had received six cycles of treatment and at least two postbaseline bone marrow assessments or had an end-of-study assessment at any timepoint. In the interim analysis, the null hypothesis was rejected if one-sided *P* < 0.00625. In the case of failure of the interim analysis, the final analysis would be tested at a one-sided alpha level of 0.02178. Two-sided 95% confidence intervals were based on the exact binomial distribution (Clopper–Pearson method). Time-to-event outcomes (DOR, PFS and OS) were determined using the Kaplan–Meier method, and estimates were computed using Greenwood’s formula. Summary statistics are presented for time to response. Maximum percentage baseline reduction in clinicopathological measures of response (bone marrow mast cells, serum tryptase, *KIT* D816V VAF and spleen volume) was based on patients with both baseline (last observation before the date of the first dose of avapritinib, including pre-dose assessments on this date) and at least one baseline assessment. The mean change from baseline AdvSM–SAF TSS to C11D1 was tested against the null hypothesis of ≥0. If one-sided, one-sample *t*-test *P* < 0.025, the null hypothesis was rejected. Summary statistics and change from baseline are presented for PGIS and QoL assessment by EORTC–QLQ–C30.

All safety analyses, QoL outcomes and secondary analyses were evaluated in the safety population, comprising all enrolled patients. All statistical analyses were conducted using SAS v.9.4 or higher.

### Trial oversight and review

The trial was designed by the sponsor (Blueprint Medicines Corporation) and trial investigators. The full protocol was approved by the institutional review board (IRB) or independent ethics committee (IEC) of each participating center: St Michael’s Hospital Research Ethics Board, Toronto, Canada; East of England – Cambridge South Research Ethics Committee, Nottingham, UK; University of Pennsylvania Office of Regulatory Affairs, PA, USA; Dana-Farber Cancer Institute, Office for Human Research Studies, MA, USA; University of Utah IRB, UT, USA; University of Michigan Medical School Institutional Review Board, MI, USA; MD Anderson Cancer Center, Office of Protocol Research, TX, USA; Stanford University, Research Compliance Office, CA, USA; Columbia University Medical Center IRB, NY, USA; Western IRB, WA, USA; Rush University Medical Center IRB, IL, USA; Washington University, Human Research Protocol Office, MO, USA; Roswell Park IRB, NY, USA; Region Syddanmark, Vejle, Denmark; Comité de protection des personnes Sud Est 1, Saint-Étienne, France; Ethikkommission II der Universität Heidelberg, Medizinische Fakultät, Mannheim, Germany; Medical Ethics Review Board, Groningen, the Netherlands; Comitato Etico Campania Sud – ASL; Napoli 3 Sud, Italy; Comitato Etico Area Vasta Centro, Florence, Italy; CEIC Hospital Universitari Pare Tau Ii – Oficina de Recerca, Barcelona, Spain; REK SØR/ØST, Oslo, Norway; and Independent Bioethics Committee for Scientific Research at the Medical University of Gdansk, Gdansk, Poland. The trial was conducted in accordance with the Declaration of Helsinki, International Conference on Harmonisation guidelines for Good Clinical Practice and local regulations. All patients provided written informed consent. Participants were not compensated, except for reimbursement of reasonable travel expenses. Each local IRB/IEC was notified based on local regulations (where required by the IRB/IEC) of all serious, unexpected adverse drug reactions involving risk to human patients. The sponsor and authors jointly collected and analyzed the data. All authors had access to all data, reviewed and provided critical input to the manuscript and made the decision to submit it for publication. All authors vouch for the validity of the trial results and adherence to the protocol.

### Reporting Summary

Further information on research design is available in the Nature Research Reporting Summary linked to this article.

## Online content

Any methods, additional references, Nature Research reporting summaries, source data, extended data, supplementary information, acknowledgements, peer review information; details of author contributions and competing interests; and statements of data and code availability are available at 10.1038/s41591-021-01539-8.

## Supplementary information

Supplementary Information Supplementary Tables 1 and 2.

Reporting Summary

## Data Availability

The anonymized derived data from this trial that underlie the results reported in this article will be made available, beginning 12 months and ending 5 years after this article’s publication, to any investigators who sign a data access agreement and provide a methodologically sound proposal to medinfo@blueprintmedicines.com. The trial protocol will also be made available, as will a data fields dictionary.
