# Supplementary material for: Efficacy and safety of avapritinib in advanced systemic mastocytosis: interim analysis of the phase 2 PATHFINDER trial
Source: Nat Med. 2021 Dec 6;27(12):2192–9. doi: 10.1038/s41591-021-01539-8 (PMC8674139; doi:10.1038/s41591-021-01539-8)
Supplement: Supplementary file 1 — Supplementary Tables 1 and 2. [file 41591_2021_1539_MOESM1_ESM.pdf]

nature portfolio

https://doi.org/10.1038/s41591-021-01539-8

Supplementary information

Efficacy and safety of avapritinib in advanced systemic mastocytosis: interim analysis of the phase 2 PATHFINDER trial

In the format provided by the authors and unedited

Efficacy and Safety of Avapritinib in Advanced Systemic Mastocytosis: Interim Analysis of the Phase 2 PATHFINDER trial

Supplementary Information

Table of Contents

Supplementary Table 1 | mIWG-MRT-ECNM Definition of Evaluable Organ Damage (C-Findings) 2

Supplementary Table 2 | mIWG-MRT-ECNM Response Criteria in Advanced Systemic Mastocytosis 4

Supplementary Table 1 | mIWG-MRT-ECNM Definition of Evaluable Organ Damage (C-Findings)

**Non-hematologic C-Findings**	
Ascites or pleural effusion	Symptomatic ascites or pleural effusion requiring medical intervention such as:<br>1. Use of diuretics (Grade 2) or<br>2. ≥2 therapeutic paracenteses *or* thoracenteses (Grade 3) ≥28 days apart over 12 weeks before C1D-8 with 1 procedure performed during the 6 weeks before C1D-8
Liver function abnormalities	Grade ≥2 abnormalities in direct bilirubin (>1.5 × ULN), AST (>3.0 × ULN), ALT (>3.0 × ULN) or AP (>2.5 × ULN) in the presence of:<br>• Ascites *and/or*<br>• Clinically relevant portal hypertension, *and/or*<br>• Liver MC infiltration that is biopsy-proven *or*<br>• No other identified cause of abnormal liver function
Hypoalbuminemia	Grade ≥2 hypoalbuminemia (<3.0 g/dL)
Splenomegaly	A spleen that is palpable ≥5 cm below the left costal margin
**Hematologic C-Findings**	
ANC	Grade ≥3 ANC (<1.0 × 10^9/L)
Anemia (transfusion-independent)	Grade ≥2 Hgb (<10 g/dL)
Anemia (transfusion-dependent)	• Transfusion of ≥6 units PRBCs in the 12 weeks before C1D-8 *and*<br>• Most recent transfusion occurring during the 4 weeks before C1D-8 *and*<br>• Transfusions administered for Hgb ≤8.5 g/dL *and*

	• Reason for transfusions is not bleeding, hemolysis, or therapy-related
Thrombocytopenia (transfusion-independent)	Grade ≥2 thrombocytopenia (<75 × 10^9/L)
Thrombocytopenia (transfusion-dependent)	• Transfusion of ≥6 units of apheresed platelets (or ≥6 pools of random donor or buffy coat) 12 weeks before C1D-8 *and*<br>• ≥2 units transfused 4 weeks before C1D-8 *and*<br>• Transfusions administered for platelet count <20 × 10^9/L

ALT, alanine aminotransferase; ANC, absolute neutrophil count; AP, alkaline phosphatase; AST, aspartate aminotransferase; C, cycle; D, day; Hgb, hemoglobin; mIWG-MRT-ECNM, modified International Working Group-Myeloproliferative Neoplasms Research and Treatment; MC, mast cell; PBRC, packed red blood cell; ULN, upper limit of normal.

≥1 C-finding is required for eligibility. Grade is based on the Common Terminology Criteria for Adverse Events, Version 5.0.

Supplementary Table 2 | mIWG-MRT-ECNM Response Criteria in Advanced Systemic Mastocytosis

Response	Criteria for Response
CR	Requires all four of the following criteria, and response duration must be ≥12 weeks:<br>• No presence of compact neoplastic MC aggregates in the BM or other biopsied extracutaneous organ<br>• Serum tryptase level <20 ng/mL<br>• Peripheral blood count remission defined as:<br>○ ANC ≥1 × 10^9/L with normal differential (absence of neoplastic MCs and blasts <1%) *and*<br>○ Platelet count ≥100 × 10^9/L *and*<br>○ Hgb level ≥11 g/dL<br>• Complete resolution of palpable hepatosplenomegaly and all biopsy-proven or suspected SM-related organ damage (C-findings)
CRh	Requires all criteria for CR be met and response duration must be ≥12 weeks; however, patient may have residual cytopenias. The following minimum recovery of peripheral blood counts is required:<br>• ANC >0.5 × 10^9/L with normal differential (absence of neoplastic MCs and blasts <1%) *and*<br>• Platelet count >50 × 10^9/L *and*<br>• Hgb level >8.0 g/dL
PR	Requires all three of the following criteria, and response duration must be ≥12 weeks, in the absence of CR/CRh and PD:<br>• Reduction by ≥50% in neoplastic MCs in the BM *and/or* other extracutaneous

	organ at biopsy demonstrating eligible SM-related organ damage<br>• Reduction of serum tryptase level by ≥50%<br>• Resolution of one or more biopsy-proven or suspected SM-related organ damage (C-findings)	
Clinical improvement	• Response duration must be ≥12 weeks<br>• Requires one or more of the non-hematologic and/or hematologic response criteria to be fulfilled in the absence of CR, CRh, PR, or PD	
SD	Not meeting criteria for CR/CRh, PR, CI, or PD	
PD	Requires ≥1 element from the criteria below; duration must be ≥4 weeks:	
	**Baseline**	**Post Baseline**
	Any Grade 2 non-hematologic organ damage	• Worsening by 1 grade *and*<br>• Minimum 100% increase (doubling) of laboratory abnormality
	Grade ≥2 albumin	• Worsening by 1 grade *and*<br>• Decrease by ≥0.5 g/dL
	Grade ≥3 non-hematologic organ damage	Minimum 100% increase (doubling) of laboratory abnormality
	Grade ≥2 transfusion-independent anemia or thrombocytopenia	New transfusion dependence for an 8-week period of ≥4 units of PRBCs or platelets

ANC, absolute neutrophil count; BM, bone marrow; CR, complete remission; CRh, complete remission with partial recovery of peripheral blood counts; Hgb, hemoglobin; mIWG-MRT-ECNM, modified International Working Group-Myeloproliferative Neoplasms Research and Treatment; MC, mast cells; PBRCs, packed red blood cells; PD, progressive disease; PR, partial remission; SD, stable disease; SM, systemic mastocytosis.
