# Supplementary material for: Efficacy and safety of avapritinib in advanced systemic mastocytosis: interim analysis of the phase 2 PATHFINDER trial
Source: Nat Med. 2021 Dec 6;27(12):2192–9. doi: 10.1038/s41591-021-01539-8 (PMC8674139; doi:10.1038/s41591-021-01539-8)
Supplement: Supplementary file 2 — Reporting Summary [file 41591_2021_1539_MOESM2_ESM.pdf]

nature research

Corresponding author(s): Jason Gotlib

Last updated by author(s): Sep 2, 2021

Reporting Summary

Nature Research wishes to improve the reproducibility of the work that we publish. This form provides structure for consistency and transparency in reporting. For further information on Nature Research policies, see our Editorial Policies and the Editorial Policy Checklist.

Statistics

For all statistical analyses, confirm that the following items are present in the figure legend, table legend, main text, or Methods section.

n/a	Confirmed	
☐	☒	The exact sample size (*n*) for each experimental group/condition, given as a discrete number and unit of measurement
☒	☐	A statement on whether measurements were taken from distinct samples or whether the same sample was measured repeatedly
☐	☒	The statistical test(s) used AND whether they are one- or two-sided<br>*Only common tests should be described solely by name; describe more complex techniques in the Methods section.*
☒	☐	A description of all covariates tested
☒	☐	A description of any assumptions or corrections, such as tests of normality and adjustment for multiple comparisons
☐	☒	A full description of the statistical parameters including central tendency (e.g. means) or other basic estimates (e.g. regression coefficient) AND variation (e.g. standard deviation) or associated estimates of uncertainty (e.g. confidence intervals)
☐	☒	For null hypothesis testing, the test statistic (e.g. *F*, *t*, *r*) with confidence intervals, effect sizes, degrees of freedom and *P* value noted<br>*Give P values as exact values whenever suitable.*
☒	☐	For Bayesian analysis, information on the choice of priors and Markov chain Monte Carlo settings
☒	☐	For hierarchical and complex designs, identification of the appropriate level for tests and full reporting of outcomes
☒	☐	Estimates of effect sizes (e.g. Cohen's *d*, Pearson's *r*), indicating how they were calculated

Our web collection on statistics for biologists contains articles on many of the points above.

Software and code

Policy information about availability of computer code

Data collection	No software used
Data analysis	SAS version 9.4

For manuscripts utilizing custom algorithms or software that are central to the research but not yet described in published literature, software must be made available to editors and reviewers. We strongly encourage code deposition in a community repository (e.g. GitHub). See the Nature Research guidelines for submitting code & software for further information.

Data

Policy information about availability of data

All manuscripts must include a data availability statement. This statement should provide the following information, where applicable:

- Accession codes, unique identifiers, or web links for publicly available datasets
- A list of figures that have associated raw data
- A description of any restrictions on data availability

The anonymized derived data from this trial that underlie the results reported in this article will be made available, beginning 12 months and ending 5 years after this article’s publication, to any investigators who sign a data access agreement and provide a methodologically sound proposal to medinfo@blueprintmedicines.com. The trial protocol will also be made available, as will a data fields dictionary.

Field-specific reporting

Please select the one below that is the best fit for your research. If you are not sure, read the appropriate sections before making your selection.

☒ Life sciences ☐ Behavioural & social sciences ☐ Ecological, evolutionary & environmental sciences

For a reference copy of the document with all sections, see nature.com/documents/nr-reporting-summary-flat.pdf

Life sciences study design

All studies must disclose on these points even when the disclosure is negative.

Sample size	The interim analysis wasperformed when 32 patients in Cohort 1 had received 6 cycles of treatment and ≥ 2 post-baseline bone marrow assessments or had an end-of-study assessment at any timepoint. The null hypothesis ORR of 28% was based on the ORR per IWG-MRT-ECNM criteria for midostaurin.9 In the interim analysis, the null hypothesis rejected if the 1-sided P < .00625. In the case of failure of the interim analysis, a sample size of approximately 63 patients in Cohort 1 at final analysis was determined to have 93.5% power at the 1-sided significance level of 0.025 for the null hypothesis ORR of 28% versus the alternative hypothesis ORR of 50%.
Data exclusions	No data exclusions
Replication	N/A. This was an interim analysis of a non-randomized Phase 2 clinical study in patients with advanced systemic mastocytosis.
Randomization	Single-arm study - no randomization
Blinding	Single-arm study - no blinding

Reporting for specific materials, systems and methods

We require information from authors about some types of materials, experimental systems and methods used in many studies. Here, indicate whether each material, system or method listed is relevant to your study. If you are not sure if a list item applies to your research, read the appropriate section before selecting a response.

Materials & experimental systems

n/a	Involved in the study	
☒	☐	Antibodies
☒	☐	Eukaryotic cell lines
☒	☐	Palaeontology and archaeology
☒	☐	Animals and other organisms
☐	☒	Human research participants
☐	☒	Clinical data
☒	☐	Dual use research of concern

Methods

n/a	Involved in the study	
☒	☐	ChIP-seq
☒	☐	Flow cytometry
☒	☐	MRI-based neuroimaging

Human research participants

Policy information about studies involving human research participants

Population characteristics	N/A - no covariate analyses conducted. The patient demographics are described in full in the manuscript (Table 1).<br>Of all 62 enrolled patients, 28 (45%) were female and 34 (55%) were male. The median participant age was 69 years (range 31–88 years).
Recruitment	Patients were recruited by participating investigators. The Investigator at each center ensured that the patients were given full and adequate oral and written information about the nature, purpose, possible risk, and benefit of the study. Patients were also notified that they were free to discontinue from the study at any time. Patients were given the opportunity to ask questions and allowed time to consider the information provided. Inclusion and exclusion criteria are defined in full in tables in the manuscript Supplementary Information. All patients provided written informed consent. Participants were not compensated, except for the reimbursement of reasonable travel expenses.<br><br>Patients were enrolled across 18 study centers in North America and Europe. Due to the geographical distribution of the study centers, participants may not represent the global general population. No other bias emerging from recruitment is expected.
Ethics oversight	The full protocol was approved by the institutional review board or independent ethics committee of each participating center, and written informed consent was obtained from all participants.

Note that full information on the approval of the study protocol must also be provided in the manuscript.

Clinical data

Policy information about clinical studies

All manuscripts should comply with the ICMJE guidelines for publication of clinical research and a completed CONSORT checklist must be included with all submissions.

Clinical trial registration	ClinicalTrials.gov: PATHFINDER study (NCT03580655)
Study protocol	Requests for the study protocol should be directed to the Sponsor via medinfo@blueprintmedicines.com
Data collection	Patients recruited and data collected at hospitals and medical centers between November 2018 and June 2020 (data cut-off): 28 patients were enrolled at 10 sites in North America, and 34 patients at 8 sites in Europe.
Outcomes	Primary endpoint: overall response rate as assessed by mIWG-MRT-ECNM criteria. Key secondary endpoint was change in total symptom score per AdvSM-SAF. Other secondary endpoints: duration of response, changes in mast cell burden (number of bone marrow mast cells, serum tryptase levels, KIT D816V variant allele fraction, and spleen volume), and safety per adverse events (National Cancer Institute Common Terminology Criteria for AEs, version 5.0), changes in vital signs, electrocardiograms, and laboratory testing.
